# Creation of an anti-imaging system using binary optics

**DOI:** 10.1038/srep33064

**Published:** 2016-09-13

**Authors:** Haifeng Wang, Jian Lin, Dawei Zhang, Yang Wang, Min Gu, H. P. Urbach, Fuxi Gan, Songlin Zhuang

**Affiliations:** 1 Shanghai Key Laboratory of Modern Optical System, Optical instruments and Systems Engineering Research Center of Ministry of Education, School of Optical-Electrical and Computer Engineering, University of Shanghai for Science and Technology, 200093, Shanghai, China; 2 Shanghai Institute of Optics and Fine Mechanics, Chinese Academy of Sciences, Qinghe Road No. 390, Jiading, Shanghai 201800, China; 3 Artifical-Intelligence Nanophotonics Laboratory, School of Science, RMIT University, Melbourne, Victoria 3001, Australia; 4 Optics Research Group, Department of Imaging Physics, Delft University of Technology, Lorentzweg 1, 2628 CJ Delft, The Netherlands

## Abstract

We present a concealing method in which an anti-point spread function (APSF) is generated using binary optics, which produces a large-scale dark area in the focal region that can hide any object located within it. This result is achieved by generating two identical PSFs of opposite signs, one consisting of positive electromagnetic waves from the zero-phase region of the binary optical element and the other consisting of negative electromagnetic waves from the pi-phase region of the binary optical element.

An imaging system forms an image by detecting the reflection, scattering or transmission of the electromagnetic/acoustic field of an object. Therefore, to hide an object from detection, one must prevent the detection of reflection, scattering or transmission from an object^1^. In a practical situation in which the detection system in question is a satellite or radar, which is typically located above the object of interest, one could design a special cover, such as a ground-plane carpet^2^,^3^,^4^,^5^,^6^,^7^,^8^,^9^. An alternative solution is to design a special shell^10^,^11^,^12^,^13^,^14^,^15^,^16^,^17^ that causes all incoming light to propagate within the shell and bypass the center region and then to restore the incoming light field on the opposite side^10^,^11^,^12^,^13^,^14^,^15^,^16^,^17^. This approach is actually an anti-imaging technique that attempts to de-resolve an object rather than to resolve it. Here, we wish to demonstrate that an imaging lens can be turned into an anti-imaging lens to hide a given target using binary optics to generate an anti-PSF (APSF) in the focal region of a lens. In effect, such a binary optics system changes the PSF of the lens into an APSF, resulting in a large dark area in the focal region. Any object located inside this dark area does not scatter or reflect any electromagnetic waves; all electromagnetic waves bypass the object as if it does not exist.

## Results

**Figure 1:**
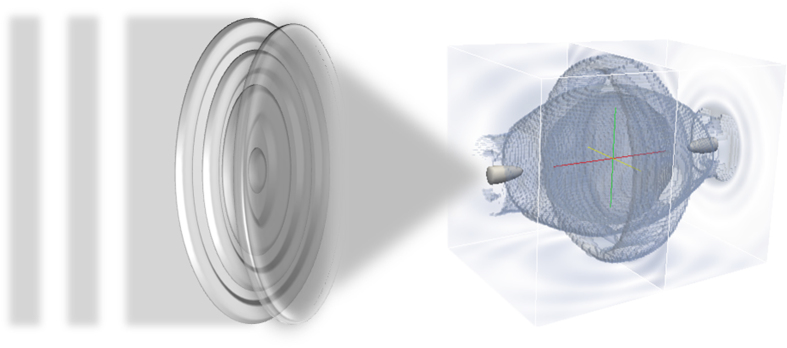
Schematic of an anti-imaging setup consisting of a concentric multi-belt binary optical element and a focusing lens. Radar waves from the left side go through the binary element and lens, which bypass a specific 3D region surrounding the focal point, forming a free space of 3D concealment.

**Figure 2:**
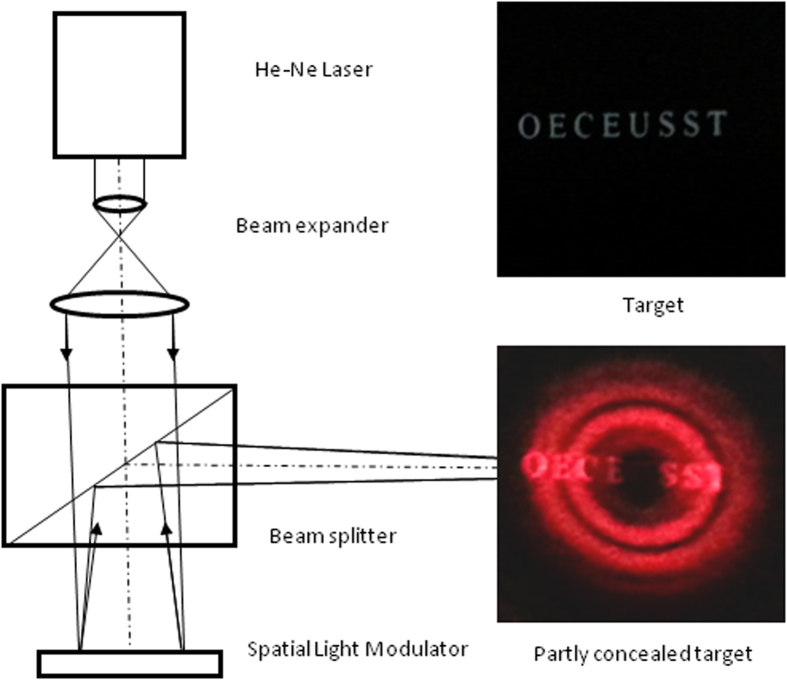
Experimental verification of the concealing capability of the anti-imaging system. A collimated laser beam projects onto a reflective phase-type SLM, which generates a phase distribution representing the combination of the binary optics and the thin lens shown in Fig. 1. The reflected beam from the SLM forms an APSF in the focal region. The target consists of the white string “OECEUSST” with a font size of 7 on black background paper. When the APSF is projected onto the target, the letter “U” in the string located in the center of the APSF is concealed.

As shown in Fig. 1, to prevent the detection of an object by a probing electromagnetic plane wave from a far-away radar system, we generate an APSF using a binary optical element and a lens, and we place an object inside this APSF. This APSF ensures that all electromagnetic waves will bypass the target that we wish to hide. The binary optical element consists of a multi-belt phase element with a phase shift of 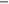 between each pair of adjacent belts. In our experiment, we use a phase-type spatial light modulator (SLM) to generate a reflective focusing lens with an effective NA of 0.001. Then, we combine the phase of the binary optical element with it to modulate a laser beam, as shown in Fig. 2. The parameters of the binary optical element are shown in equation (7). The wavelength of the laser is 633 nm. An APSF is generated in the focal region of the effective lens. To show the concealing capability of this APSF, we print the white characters “OECEUSST” with font size of 7 on black background paper so that the characters will stand out if they are probed by the laser beam. Then, we place this paper in the APSF. As shown in Fig. 2, the character “U” in the string “OECEUSST” is invisible because the entire light field bypasses the center region in the APSF, so that no light field is scattered when an object is put there, as if it does not exist at all. The font size of 7 is 1.94 mm, corresponding to approximately 3064 wavelengths of the light. If we use a 1.0 THz wave, this APSF can hide an object approximately 1.0 meter wide. Because the THz waves range from 0.1 THz to 10 THz, this APSF can hide an object of 0.1 meter to 10 meters wide. The size of the APSF can be increased or decreased by changing the numerical aperture (NA) of the focusing lens.

## Methods

**Figure 3:**
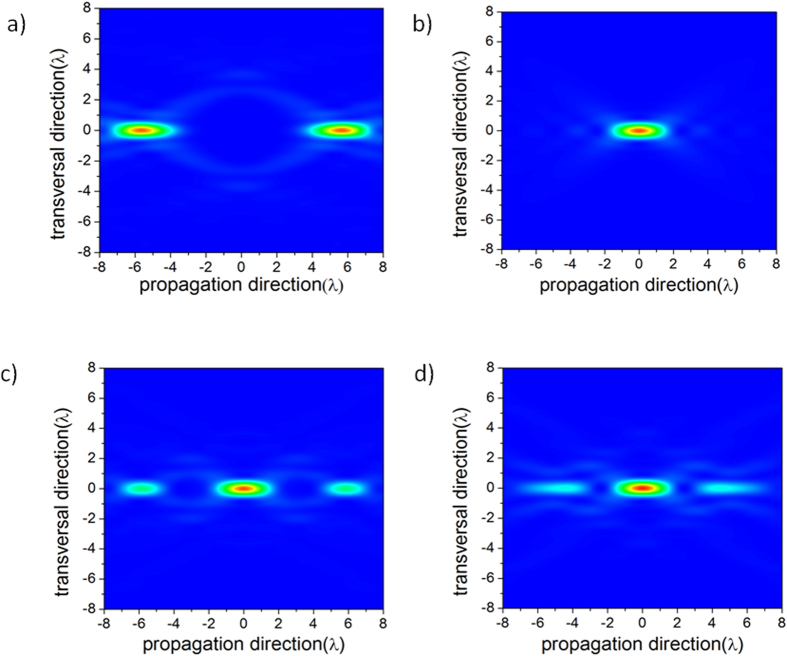
The simulated APSF, PSF, positive PSF and negative PSF in the focal region of a lens with NA = 0.8. (**a**) The APSF generated by the lens and a binary optical element consisting of zero and pi phases. (**b**) The PSF generated by the lens. (**c**) The positive PSF, consisting of positive electromagnetic waves, generated by the zero-phase region of the binary optical element. (**d**) The negative PSF, consisting of negative electromagnetic waves, generated by the pi-phase region of the binary optical element.

The radius of each belt is obtained by finding the local minimum of the light-field intensity in a 3D space near the focal region of the imaging lens. This process can be simplified by finding the local minimum with a flat response along the optical axis^18^,^19^,^20^,^21^,^22^ and along the transversal direction. Binary-optics technology is often applied to obtain super-resolution and produce non-diffraction beams^18^,^19^,^20^, generate longitudinally polarized light^21^, create large-scale single-beam optical traps for atoms^22^ and extend the depth of field for stimulated-emission depletion fluorescence microscopy (STED)^23^. Here, this technology is adopted to disable the imaging capability of a radar system. As an example, we choose a lens with a numerical aperture (NA) of 0.8, and the simulated APSF is shown in Fig. 3a. It is clear that the field in the focal region essentially reaches zero within 4 wavelengths. Therefore, any object located within that region is invisible because it produces no scattering. For comparison, the simulated PSF of the imaging system without the binary optical elements, which is much smaller than the APSF, is calculated and plotted in Fig. 3b. Any object that might be detected by the radar system can now be hidden in the APSF region.

This anti-imaging system was designed using the vector focusing method^18^,^19^,^20^,^21^. When a linearly polarized light field is focused by a lens, the light field in the focal region is expressed as follows:


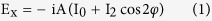



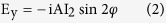



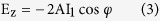


where


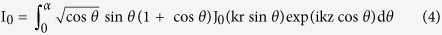



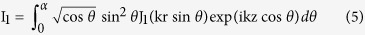



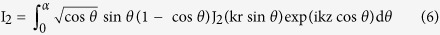


Here, *α* = arcsin(*NA*) denotes the largest focusing angle, *φ* represents the azimuthal angle, *k* is the wave number, and *J*_*n*_(*n* = 0, 1, 2) are the *n*th-order Bessel functions. The constant *A* is defined as *A* = *πl*_0_ *f*/*λ*, where *l*_0_ = 1 indicates uniform illumination and *f* is the focal length. The field in the APSF shown in Fig. 3a is calculated by superposing the fields originating from the various belts of the binary optical element^18^,^19^,^20^,^21^,^22^,^23^,^24^, and the optimization process involves finding a series of radius values for the binary optics with the goal of obtaining a constant zero axial and radial intensity within a certain region^22^. As an example, we select a lens with NA = 0.8; then, the corresponding series of radius values for the binary optics system is found to be as follows:


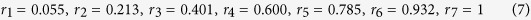


The APSF extends approximately 4 wavelengths along the transversal direction and 4 wavelengths along the axial direction. The size of the dark region (APSF) can be adjusted by choosing lenses with varying NA values. The relationship between the size of the dark region and the NA is as follows: in the transversal direction, the APSF size is inversely proportional to the NA, i.e., ~1/NA, while in the beam-propagation direction, it is inversely proportional to the square of the NA, i.e., ~1/(NA)^2.

Therefore, by choosing different NA values and wavelengths of electromagnetic waves, different sizes of APSF can be achieved, as shown in Table 1. It is clear that, for visible light, large APSFs cannot be generated using this method; therefore, it is not possible to hide large objects from visible light^25^. However, for radar waves, from millimeter waves to meter waves, larger APSFs of a few meters to tens of meters can be achieved, which makes it possible to hide bulky objects. The APSF shown in Fig. 3a is actually the result of combining the positive PSF shown in Fig. 3c and the negative PSF shown in Fig. 3d, where the positive PSF is generated by the zero-phase region of the binary optical element, and the negative PSF is generated by the pi-phase region of the binary optical element^20^,^21^,^22^. The positive light in the positive PSF and the negative light in the negative PSF offset one another, resulting in a dark region^22^. We first performed large-scale dark-spot generation in 2001 using positive and negative light from a binary optical element to trap atoms^22^; now, this technology has found an application in anti-imaging.

In fact, when the NA of the focusing lens is decreased, the sizes of both the positive PSF and the negative PSF increase. In the extreme case, when the NA of the focusing lens is zero, both the positive PSF and the negative PSF become plane waves; then, it is possible to use an anti-radar system to generate negative radar waves to protect a given target. For example, suppose a probing radar wave is sent toward an object. This wave is captured by an anti-radar device, which generates an anti-radar wave and projects it toward the object, so that the radar wave and the anti-radar wave offset one another near the object, causing a dark region surrounding the object and thereby hiding it.

In summary, we have proposed an anti-imaging technique based on an APSF generated using binary optics. The binary optics system is used to generate two identical PSFs of opposite signs, one consisting of positive electromagnetic waves and the other consisting of negative electromagnetic waves. The positive electromagnetic waves originate from the zero-phase region of the binary optical element, and the negative electromagnetic waves originate from the pi-phase region of the binary optical element. The combination of the positive and negative electromagnetic waves in the focal region creates a large dark region, thus forming an APSF. The APSF can be used to hide objects meters long when a low-NA focusing lens and radar waves are used. We can also treat the positive electromagnetic waves as the waves from a probing radar system and the negative electromagnetic waves as the ones from an anti-radar system; the anti-radar waves can be used to offset the radar waves in a certain region to protect a specific target. Currently, binary optics technology has found applications in achieving super-resolution^1^,^18^,^19^,^20^,^21^,^26^, producing non-diffraction beams^18^,^19^,^20^, generating longitudinally polarized light^21^, creating large-scale dark-spot optical traps for atoms^22^, extending the depth of field for stimulated-emission depletion fluorescence microscopy (STED)^23^ and, now, anti-imaging. We expect that this technology may yet be applied to still more fields^27^. Dark spots of submicron size have also been generated through structured illumination^28^,^29^ or thermal effects in stacks^30^, which can be used in concealment^29^ or optical tweezing^30^. It would be interesting to combine the index matching technique with binary optics^31^,^32^, which may make the binary optics system itself invisible.

## Additional Information

**How to cite this article**: Wang, H. *et al.* Creation of an anti-imaging system using binary optics. *Sci. Rep.* **6**, 33064; doi: 10.1038/srep33064 (2016).

## Figures and Tables

**Table 1:** The APSF sizes for different NA values and wavelengths.

NA	wavelength	APSF size (transversal)	APSF size (axial)
0.8	1.0 m	4.0 m	4.0 m
0.1	1.0 m	32 m	256 m
0.001	633 nm	2 mm	1.620 m
0.001	2000 nm	6.4 mm	5.118 m
0.001	0.3 mm	960 mm	768 m
